# Wearable Urban Mobility Assistive Device for Visually Impaired Pedestrians Using a Smartphone and a Tactile-Foot Interface

**DOI:** 10.3390/s21165274

**Published:** 2021-08-04

**Authors:** Ricardo Tachiquin, Ramiro Velázquez, Carolina Del-Valle-Soto, Carlos A. Gutiérrez, Miguel Carrasco, Roberto De Fazio, Andrés Trujillo-León, Paolo Visconti, Fernando Vidal-Verdú

**Affiliations:** 1 Facultad de Ingeniería, Universidad Panamericana, Josemaría Escrivá de Balaguer 101, Aguascalientes 20290, Mexico; rtachiquin@up.edu.mx; 2 Facultad de Ingeniería, Universidad Panamericana, Álvaro del Portillo 49, Zapopan 45010, Mexico; cvalle@up.edu.mx; 3 Facultad de Ciencias, Universidad Autónoma de San Luis Potosí, Av. Chapultepec 1570, Privadas del Pedregal, San Luis Potosí 78295, Mexico; cagutierrez@fc.uaslp.mx; 4 Facultad de Ingeniería y Ciencias, Universidad Adolfo Ibañez, Av. Diagonal las Torres 2640, Santiago 7941169, Chile; miguel.carrasco@uai.cl; 5 Department of Innovation Engineering, University of Salento, Via per Monteroni, 73100 Lecce, Italy; roberto.defazio@unisalento.it (R.D.F.); paolo.visconti@unisalento.it (P.V.); 6 Departamento de Electrónica, Universidad de Málaga, Andalucía Tech, Campus de Teatinos, 29071 Málaga, Spain; atrujilloleon@uma.es (A.T.-L.); fvidal@uma.es (F.V.-V.)

**Keywords:** assistive technology (AT), augmented GPS (A-GPS), navigation mobile app, outdoor orientation, tactile-foot interface, visually impaired pedestrians, urban mobility

## Abstract

This paper reports on the progress of a wearable assistive technology (AT) device designed to enhance the independent, safe, and efficient mobility of blind and visually impaired pedestrians in outdoor environments. Such device exploits the smartphone’s positioning and computing capabilities to locate and guide users along urban settings. The necessary navigation instructions to reach a destination are encoded as vibrating patterns which are conveyed to the user via a foot-placed tactile interface. To determine the performance of the proposed AT device, two user experiments were conducted. The first one requested a group of 20 voluntary normally sighted subjects to recognize the feedback provided by the tactile-foot interface. The results showed recognition rates over 93%. The second experiment involved two blind voluntary subjects which were assisted to find target destinations along public urban pathways. Results show that the subjects successfully accomplished the task and suggest that blind and visually impaired pedestrians might find the AT device and its concept approach useful, friendly, fast to master, and easy to use.

## 1. Introduction

Research on devices and systems assisting the blind and visually impaired community has broadened during the last decades to become a major field of study in Assistive Technology (AT). This response is proportional to the dimension of the problem: according to the World Health Organization (WHO) 2019 World Report on Vision [1], there are about 235 million people with severe visual disability (including blindness), for which vision cannot be corrected with ocular surgery or the use of standard glasses.

AT for people who are blind or visually impaired can be classified into three major task-specific aids: reading, computer access, and mobility (Table 1).

Reading is a fundamental component of human culture. People with low vision naturally exhibit reduced reading capabilities, which deprive them in several aspects of daily life such as employment, education, and social interaction.

AT targeting reading solutions aims to make the proper adaptations to enable the access to printed material such as books, newspapers, and magazines. Two major reading approaches can be found in the literature: Braille books and audio books.

Research on AT devices addressing Braille books have explored low-cost yet competitive tactile printing alternatives to Braille embossers [2,3,4]. Proposals target the implementation of simple and affordable prototypes that users can privately own and use at home.

Audio books are perhaps the simplest and least expensive solution to the reading problem of people with low vision. However, they cannot be considered the definite reading solution for people with visual disability [5]. As the normally sighted do prefer to read a book instead of listening to the audio version, there is no reason to directly assume otherwise for blind or visually impaired people. Reading is a task that stimulates the intellectual activity, increases the literacy, and heightens the self-esteem of those with impaired vision. Unfortunately, audio books cannot provide these user-related aspects.

Technology plays a central role in all facets of modern life: instant access to information, communication, education, work, collaboration with peers, among many others. Digital technologies have been recognized as an essential tool for the human progress to the point that several countries around the globe have now incorporated laws to their legal basis regarding digital rights for their citizens. Such laws involve the right to use computers and the right to access the Internet [6].

AT addressing the issue of computer access aims to ensure these rights for people with visually disability by enabling the usage of electronic devices and digital text accessibility. Three major approaches can be distinguished in the literature: speech synthesizers, screen magnifiers, and Braille terminals.

Speech synthesizers consist of specialized software that literally read aloud the text displayed on a screen. Research on this topic has centered on achieving human-like speech synthesis systems that encompass the phonemes of a language, so that words and sentences are understandable, pleasant, and pronounced correctly (not merely the robotic voice) [7,8,9].

Screen magnifiers enlarge screen content and are mainly devoted for those who still have some degree of remnant vision. Research has focused on displaying with good quality the magnified text or image and the compatibility with the most popular computer operative systems [10,11].

The design and implementation of Braille terminals is one of the most active areas of research in AT for blind and visually impaired people.

Research has centered on exploring different actuator technologies that can meet Braille standards together with low implementation costs and scalability potential [12,13,14,15,16].

Mobility, to be understood in this context as walking, is essential to human nature. It provides the means for interacting with space and is a key element in our quality of life. Loss of mobility results in a substantial decrease of well-being. People with visual disability daily face difficulties for moving around, specifically in unfamiliar spaces and dynamic environments. Besides from the inherent challenges of traveling, they experience fear for personal safety (getting lost or injured), anxiety, and a lack of confidence when going out alone. AT addressing mobility aims to provide the tools, so that individuals with visual impairments are capable of moving in a variety of environments (static/dynamic and familiar/unfamiliar) in a safe, independent, and efficient way [17].

There are two processes involved in human mobility: sensing of the surrounding space and orientation during travel. The former refers to the ability of detecting imminent obstacles along the path and planning the strategy to overcome them. The latter refers to the knowledge of one’s location in space and the capacity to reach a destination [18].

Systems providing obstacle detection have been reported in the literature as early as the 1970s. Kay proposed back then the use of sonar technology to assist the blind and visually impaired in obstacle detection [19]. Later, ultrasonic technology replaced sonar systems. Borenstein deployed a set of ultrasonic sensors on a belt worn around the abdomen [20]. Hoyle introduced in [21] the Ultracane, a traditional white cane incorporating ultrasonic sensors. Lasers have also been used for this task. Farcy’s Teletact [22] is a pistol-like laser beam system providing obstacle detection. Dang et al. integrated a laser with a camera and an IMU to find obstacles and the distances from the user. Recently, RGB cameras and computer vision techniques have been used for sensing the surrounding space. Pissaloux et al. presented in [23] the use of miniature cameras mounted on the eyeglasses to acquire images from the space ahead of the user. Computer vision algorithms extract the free spaces from the objects blocking the user’s path.

Systems providing orientation can be classified in two: for indoors and for outdoors. To our knowledge, there is no single technology delivering user orientation for both types of environments.

Indoor orientation has been mainly addressed with beacon-based approaches, that is electronic devices that broadcast low-power signals to nearby receivers. In this context, Andò el al. reported in [24] the use of Radio Frequency Identification (RFID) tags positioned in the environment and an RFID reader to assist the navigation of visually impaired people. Similarly, Kulyukin et al. examined the use of passive RFID tags for assisted navigation [25]. Infrared (IR) sensors have also been explored. Hesch and Roumeliotis mounted an IR sensor on a white cane to estimate the position of a blind user in an indoor environment [26]. Jain used IR sensors and a smartphone for indoor wayfinding [27]. Recently, several wireless sensors technologies (such as ZigBee, Bluetooth, WiFi, etc.) have been found useful for this task [28].

Outdoor orientation aims to ease urban mobility for visually challenged people. Most devices rely on GPS (global positioning system) technology for providing the user’s position and guidance in space. GPS offers some interesting advantages: combined with cartography, it becomes a power tool for assisting mobility in urban settings. It provides real-time user location with good accuracy and step-by-step instructions to reach a destination. GPS is typically free, it is available worldwide, and requires minimal skill or effort to be used. Its main inconvenience is that it does not work well when large civil structures (such as buildings, tunnels, walls, roofs, etc.) obstruct the line of sight with the GPS satellites.

Some examples of systems exploiting GPS for the outdoor mobility of blind and visually impaired pedestrians can be found both commercially and in academia. For the former, the devices from Sendero [29] and HumanWare [30] provide solutions for reaching previously set destinations together with a description of the navigating paths (street names, intersections, points of interest, etc.). For the latter, different works exploring a variety of hardware architectures can be found in the literature [31,32,33]. A common feature across these devices is the acoustic feedback conveyed to the user: guidance is achieved through verbal instructions provided along the entire route.

Acoustic feedback poses a major inconvenience for this task [5]: In the absence of vision, visually disabled pedestrians rely on listening to environmental cues to detect vehicles, other people, situations, and potential dangers approaching. Acoustic feedback might interfere and distract them compromising their orientation, space awareness, and safety.

Haptic feedback, i.e., information displayed to the sense of touch, has been explored as well in outdoor orientation AT devices, though to a lesser extent. Pielot’s PocketNavigator [34] and Jacob’s system [35] exploit the vibrotactile feedback of smartphones to provide navigational instructions to pedestrians with low vision. Spiers et al. evaluated in [36] the haptic feedback of two interfaces, the Animotus and the Cricket, for guiding blindfolded subjects along outdoor public areas. Recently, Rodriguez et al. presented in [37] a prototype of electronic guide dog based on a low-cost commercial kinesthetic haptic device providing passive haptic feedback as the guide dog does through its leash.

A common feature of these haptic interfaces is that they demand constant hand interaction from the users for effectively conveying their feedback and for carrying the device. Nevertheless, long term hand interaction has two limitations: busy hands interfere with the comfortable manipulation of objects and pedestrians get quickly fatigated of holding/carrying the device.

This paper presents a novel mobility AT device providing user orientation in outdoor environments. The device consists of a smartphone and a wearable tactile display. The first encompasses a GPS sensor that ensures a reasonably accurate user location, tracking, and guidance in space. The latter consists of a haptic device that can be inserted into a shoe and that does not require hand interaction at all. The proposal is intended to serve as a complementary device to the white cane or guide dog, thus representing a complete mobility AT solution for blind and visually impaired pedestrians in urban settings.

The rest of the paper is organized as follows: Section 2 introduces the design concept, operation principle, and main components of the AT device. Section 3 presents an experimental evaluation of such device that demonstrates its effectiveness and shows initial feasibility of the approach. Finally, Section 4 concludes summarizing the main contributions of this work and providing its future perspectives.

## 2. Towards a Novel AT Device for Urban Mobility

### 2.1. Concept

A complete AT solution to the mobility challenges of blind and visually impaired pedestrians must try to address the two processes involved in human mobility: obstacle detection and orientation [38].

As mentioned in the previous section, technologies such as ultrasonic sensors and laser beams have been explored to detect obstacles along walking paths. Unfortunately, these technologies demand an active scanning of the environment involving continuous physical activity. Cameras do not require such a constant scanning as their field of view might cover a comprehensive space ahead of the user. However, image processing and the simplification of the gathered information to convey it to the user are certainly time-consuming operations and feedback might demand a significant cognitive effort. These shortcomings reduce the walking speed, quickly fatigue users (both mentally and physically), and limit obstacle detection AT devices from attaining significant and perceivable improvements in comparison with the primary aids.

The primary aids, i.e., the white cane and guide dog, are the most popular mobility aids for blind and low vision pedestrians. The white cane is multifunctional, robust, compact, inexpensive, and lightweight. It provides reliable obstacle detection in the 1–2 m range ahead of the user. Guide dogs are very capable and reliable for leading their owners around obstacles and for foreseeing potential dangers. They also become friends and companions. Furthermore, both are the icons of a visually challenged pedestrian, which is useful for getting assistance in our society. Given their longstanding success, one should wonder whether it would be more pertinent to design complementary AT devices to the primary aids rather than to keep trying to replace them.

Our proposal trusts the primary aids with the obstacle detection process and complements them with the orientation one.

Devoted to outdoor mobility, our proposal encompasses two main components: a smartphone and a wearable on-shoe tactile display. The reason for using a smartphone is mainly due to the possibility of exploiting its GPS sensor, Internet connectivity, and portable computing capabilities. The purpose of using an on-shoe tactile display is twofold: (1) tactile feedback is preferred over the acoustic modality to avoid distractions from the environmental sounds and (2) it ensures hands-free interaction allowing users to hold and manipulate the primary aids and other objects.

**Figure 1:**
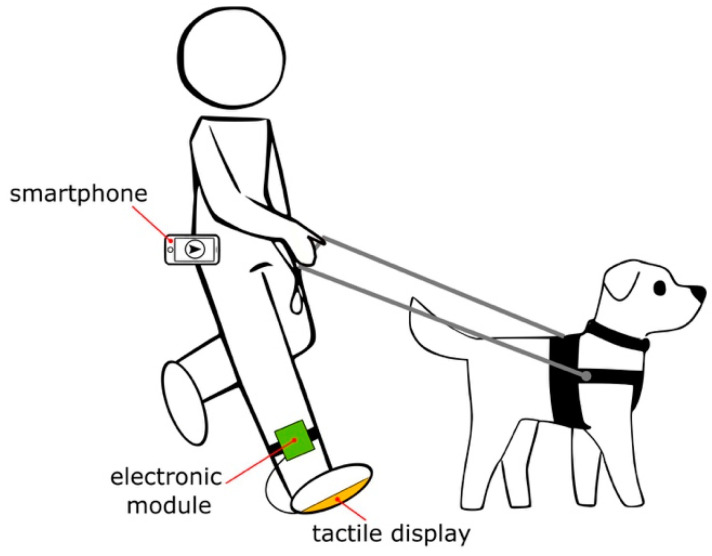
Conceptual representation of a user wearing the AT device. This concept encompasses the primary aids which are responsible for obstacle detection.

Figure 1 illustrates how a blind or low vision pedestrian would use the AT device. The smartphone is responsible for user GPS coordinate acquisition as well as for running the navigation software that generates the instructions to reach a destination. Navigational instructions are then transmitted to an electronic module that the user comfortably wears attached to the ankle. Such module translates the instructions to actuator commands and sets the tactile display accordingly. In sum, the user feels vibrations in the foot sole pointing the direction to follow.

### 2.2. Operation Principle

**Figure 2:**
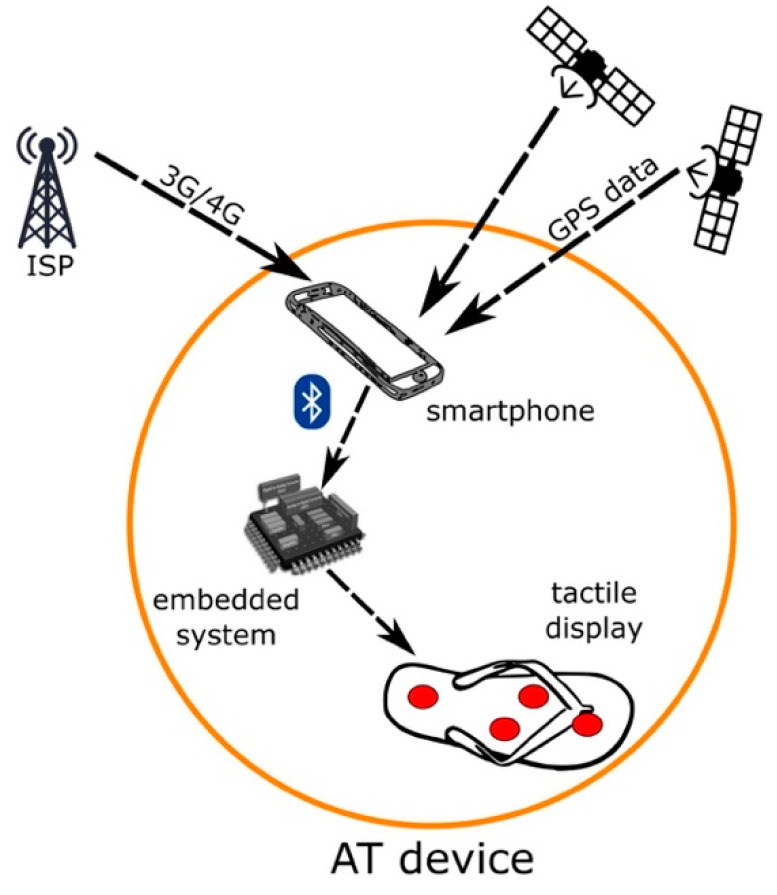
Operation principle and main components of the AT device. ISP: Internet Service Provider.

The operation principle of the proposed AT device is shown in Figure 2.

Recently, modern smartphones have become the most popular GPS receivers. Despite the 31 satellites in orbit shaping together the GNSS (global navigation satellite system) network, GPS accuracy is highly dependent on environmental factors. To increase the positioning accuracy, it is necessary to connect the smartphone to an Internet network as well.

In this prototype, we use a Samsung Galaxy S9 smartphone running Android 8.0 for GPS coordinate acquisition. Using its Internet connectivity capabilities, the smartphone connects to either the 3G or 4G networks for augmented GPS (A-GPS) accuracy.

**Figure 3:**
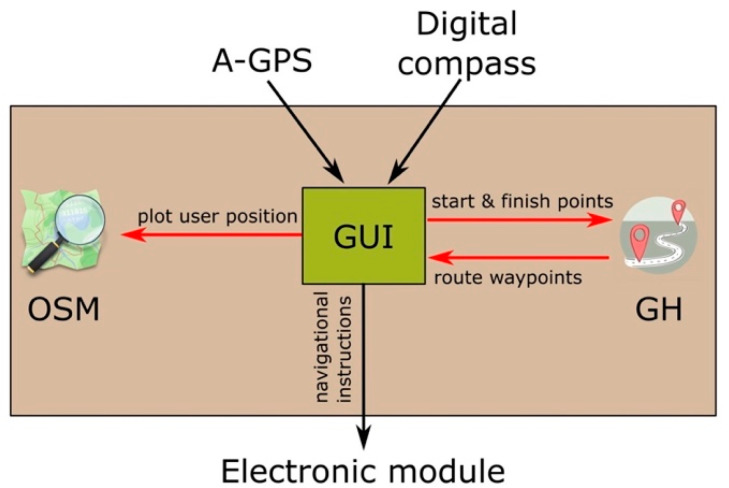
Overview of the AT device navigation software (mobile app). OSM: OpenStreetMap. GH: Graphhopper.

A dedicated Android-based app was implemented to link all the navigation software of the AT device. Such app encompasses three main elements (Figure 3): (1) The OpenStreetMap (OSM) API (application programming interface) [39], (2) the Graphhopper (GH) API [40], and (3) a self-developed script. OSM is a collaborative project that aims to create free-to-use maps of the world. Graphhopper is an open-source route planner that allows computing the shortest pedestrian route between two points. The script serves as graphical user interface (GUI), links the two former APIs, and generates the instructions for the tactile display.

Once the A-GPS coordinates have been acquired, they are plotted on the OSM map together with the user orientation signal coming from the smartphone’s digital compass. The user can then specify a destination or select it from a menu of previously saved destinations. When the destination is set, the app links to the Graphhopper API, which returns the route waypoints. The app processes these points and determines the navigational instructions to the destination. The app no longer needs to communicate with the Graphhopper API unless a route recalculation is necessary.

Instructions are then transmitted from the smartphone to the electronic module via Bluetooth. The electronic module consists of an embedded system with an ATMEL ATtiny2313 microcontroller (Microchip Technology Inc., Chandler, AZ, USA) responsible for translating the instructions to actuator commands and for transmitting these latter to the tactile display.

The app refreshes the A-GPS coordinates each 3 s or each 2 m (whatever happens first) and updates the user position in the OMS map accordingly.

The app was carefully designed seeking to ensure accessibility. For users with remnant vision, the screen content can be magnified with the app’s built-in zooming features. For blind users, the app encompasses the Android’s TalkBack screen reader [41]. Naturally, the app can also be operated by a family member, caregiver, or friend.

### 2.3. User Interface

A wearable on-shoe vibrotactile display provides the user with the necessary navigational instructions to reach the chosen destination. The novelty of this interface concept is that it provides haptic feedback to the foot.

**Figure 4:**
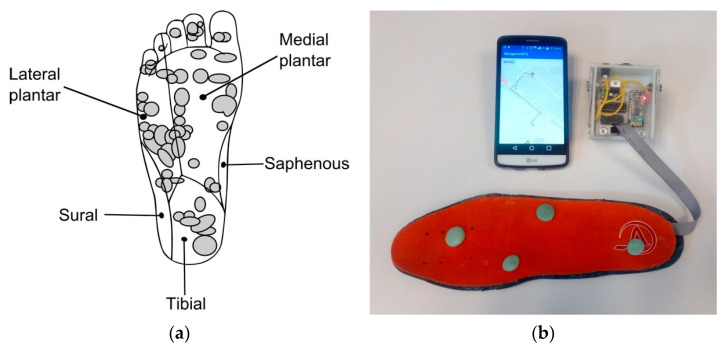
User interface: (**a**) FAI afferents on the foot sole and (**b**) prototype.

The user interface was conceived to stimulate the fast-adapting type I (FAI) afferents in the plantar surface. Four actuators (Jinlong Machinery C1030L-50, Zhejiang, China) convey vibrations to the tibial, lateral, and medial plantar areas (Figure 4a), which, according to the physiology of the foot [42], are the most sensitive to low frequency vibratory stimuli.

The four vibrating actuators were integrated in a commercially available inexpensive foam insole. An experimental characterization of the actuators [43] confirmed that they are capable of delivering axial forces up to 13 mN and vibrating frequencies between 10 and 55 Hz, demanding a maximum of 400 mW from the power source.

Dots of an epoxy paste coat the actuators’ upper surfaces ensuring a 133 mm^2^ contact area with the foot sole. The actuators’ vibrations are correctly transmitted through the epoxy paste dots. The natural absorption material properties of the foam prevent vibrations from expanding throughout the insole while cushioning the actuators against the user’s weight.

The electronic module consists of an embedded system encompassing a Bluetooth receiver (Solu JY-MCU HC-06, Shenzhen, China), the Atmel microcontroller, and the power circuitry to set the actuators. The electronic module also includes a nickel-metal hydride rechargeable battery bank (Radio Shack 23-338, Fort Worth, TX, USA) providing 6 V and 1500 mAh. This battery allows a 6 h continuous operation of the user interface. The Bluetooth receiver ensures up to a 10 m communication distance with the smartphone, which extensively covers the distance between the user’s waist and ankle (see Figure 1).

Figure 4b shows the prototype of AT device developed. The user interface is meant to be used on the right foot and is fully wearable. Note that it becomes visually unnoticeable: it is further inserted into the shoe and the user’s clothing can cover the electronic module. The approximate laboratory cost of both electronic module and user interface is USD 250. A much lower cost can be expected upon industrial mass production. A wide range of usages can then be envisaged: from an affordable-to-everyone AT device to a disposable item.

This device is the fourth version of wearable electronic on-shoe tactile displays that our team has implemented. This last version incorporates the technological improvements of the three previous developments [43,44,45].

## 3. Evaluation

Two user experiments were conducted to evaluate the performance of the AT device: navigational instruction recognition and urban mobility. Both are detailed in this section.

### 3.1. Experiment I: Navigational Instruction Recognition

The first experiment aimed at determining whether a group of voluntary subjects was capable of recognizing the navigational instructions displayed by the user interface.

#### 3.1.1. Participants

Twenty normally sighted undergraduate students (2 females, 18 males) at Universidad Panamericana (Guadalajara, Mexico) volunteered to take part in the experiment. Ages ranged from 18 to 22 years old with an average of 19. All subjects provided informed consent prior to participation according to the university ethics guidelines. No criteria were used to select them but motivation and availability. None of them reported any (known) disability in their sense of touch, feet, or cognition.

#### 3.1.2. Procedure

The user interface was inserted into a slipper (Crocs Inc., Niwot, CO, USA) and the electronic module was attached to subjects’ ankle using a hook-and-loop fastener (Velcro Co., Manchester, NH, USA). The use of socks was requested for hygiene purposes. Both slipper and user interface were cleaned with disinfecting spray after the participation of each subject.

Prior to the experiment, the subjects were unaware of any aspect concerning the task and were given general instructions. A short familiarization time with the user interface was provided (less than 5 min). During this time, the navigational instructions were displayed and explained to the subjects.

**Figure 5:**
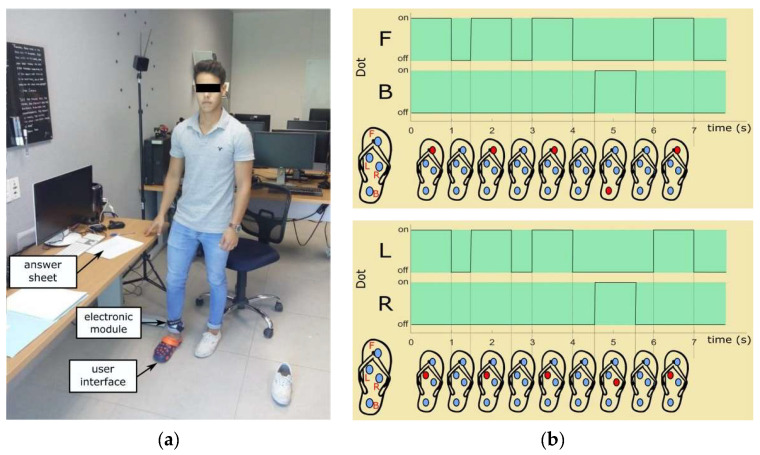
Navigational instruction recognition experiment: (**a**) setup and (**b**) vibrating patterns for (top) going forward and (down) turning left. The dot being actuated is represented in red. The whole pattern lasts 7 s.

Subjects were standing during the entire experiment. They were requested to fill an answer sheet reporting the navigational instruction perceived (Figure 5a).

A research assistant was responsible for operating the smartphone and for sending the instructions to the user interface.

#### 3.1.3. Method

Each of the four dots of the user interface represents an instruction: go forward (F), go backward (B), turn left (L), and turn right (R).

The tactile rendering method used for displaying an instruction is as follows: three consecutive short vibrations in the corresponding dot, one short vibration in the opposite dot, and again a short vibration in the corresponding dot.

Figure 5b shows, for example, the tactile patterns for going forward and for turning left. For the former, note that dot F is consecutively set three times, then dot B once, and again dot F. For the latter, dot L vibrates three times, then dot R, and again dot L.

A set of 20 instructions randomly encompassing five times each instruction was displayed during the test. Subjects could have the instruction refreshed on the interface upon request.

#### 3.1.4. Results

The results obtained from the 20 subjects are shown in the confusion matrix in Table 2. The mean recognition rates were 100% for F, 100% for B, 95% for L, and 93% for R.

These high recognition rates are mainly due to the optimized tactile rendering method shown in Figure 5b in which the opposite dot is used to display a navigational instruction as well. Such strategy provides a reliable reference to ease the identification of vibrating points when users cannot accurately discriminate which dot is actually vibrating. Contrary to confusing users, displaying both the correct and the opposite dot in the same tactile pattern eases the discrimination of the navigational instructions conveyed by the tactile-foot interface [44].

Naturally, the above rates refer to the mean scores obtained from the participants, i.e., they represent the central tendency for each navigational instruction. Additionally, it is also interesting to visualize the best and worst subject performance to appreciate how far are the limit cases and the size of the interval comprising all other subject performances.

**Figure 6:**
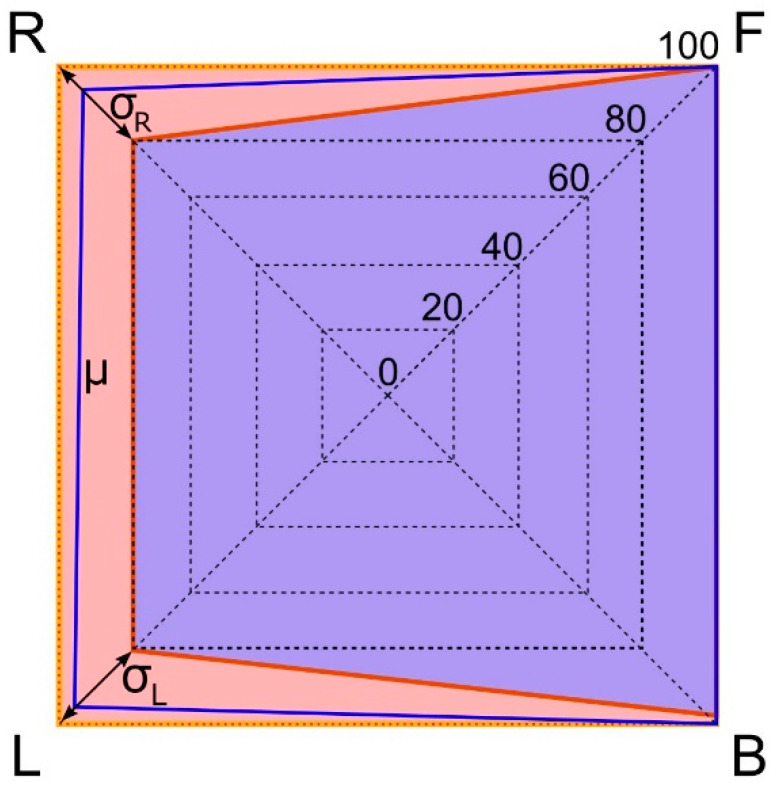
Comparative performance between the subjects with the best and worst scores. Best performance: outer orange area. Worst performance: inner purple area. Mean performance: blue line.

Figure 6 presents this analysis. The radar plot shows that the subject exhibiting the best performance obtained the 100% of correct answers for all four navigational instructions while the subject with the worst performance scored 100%, 100%, 80%, and 80% for F, B, L, and R, respectively.

Note that even the subject with the worst performance recognized two navigational instructions with no flaws. Also note that there is a narrow margin between the upper and lower performance limits. The low standard deviation values (σ_L_ = 8.88 and σ_R_ = 9.79) show that scores are clustered close to the mean (in fact, 15 out of the 20 subjects obtained perfect scores during the task). Results confirm that it is unlikely to get confused with the navigational instructions conveyed by the user interface.

### 3.2. Experiment II: Urban Mobility

The second experiment has two purposes: (1) to evaluate the performance of the AT device as a whole and (2) to determine whether it can actually assist the outdoor orientation and urban mobility of blind and visually impaired pedestrians.

#### 3.2.1. Fine-Tuning the Device

Prior to conducting any user experiment, the AT device in Figure 4b was extensively tested by the research team in urban settings to verify the correct operation of the navigation app, the position accuracy of the A-GPS, and the communication between the smartphone and the user interface.

**Figure 7:**
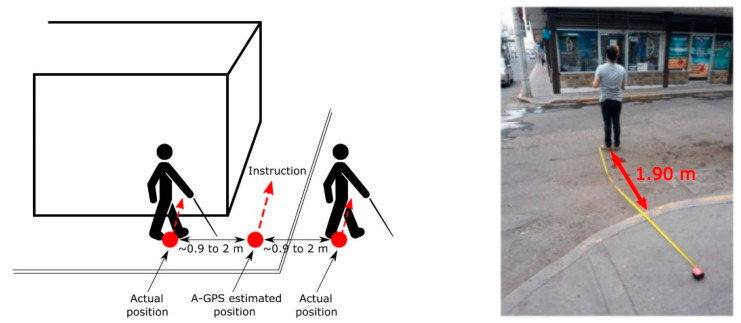
Positioning accuracy: (**left**) A-GPS enables accuracies in the range 0.9 to 2 m. (**right**) Due to this range, a navigational instruction might be displayed in the middle of the street.

In particular, for determining the position accuracy of the A-GPS, several environments in the city of Aguascalientes, Mexico were navigated. The locations in which the A-GPS indicated to display the navigational instructions were registered and compared to the corresponding user’s actual locations. Figure 7 illustrates the conclusions obtained.

Upon the use of A-GPS, it is possible to achieve a positioning accuracy of 0.9–2 m, which represents an improvement compared to using GPS alone (2 to 5 m) [46]. This means that the ideal location to display the instruction can be up to 2 m away from the point where the user interface actually displays it.

Two scenarios can be expected: (1) the instruction is displayed before the ideal point. For example, when instructed to turn, users could still encounter a wall. They would need to keep walking some steps beyond to be capable of turning. (2) The instruction is displayed after the ideal point. A more complex scenario implying that the user might no longer be on the sidewalk or even worse that he/she is already in the middle of the street (see Figure 7 right). A natural user reaction would be to stop and try to return to the sidewalk.

**Figure 8:**
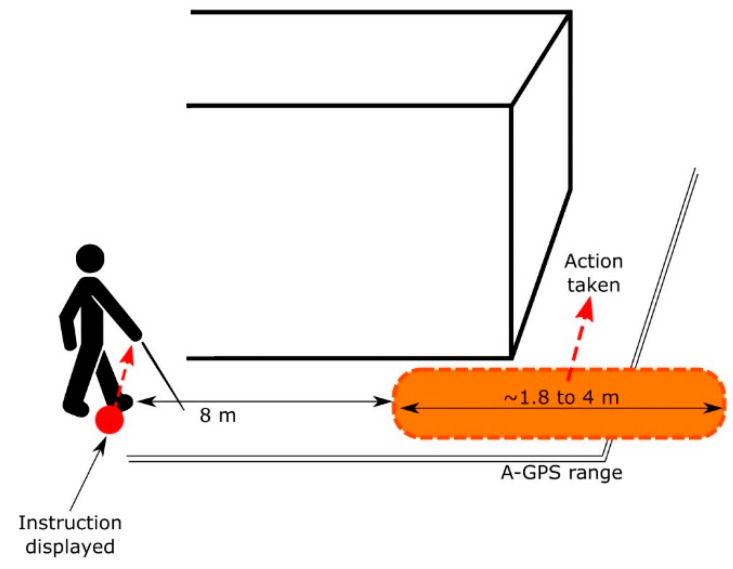
Instruction display strategy: instructions are displayed in advance reducing the possibility of conveying them down the sidewalks or when crossing the streets.

To avoid risky situations that might compromise the pedestrians’ safety, the AT device was fine-tuned to always display the instructions 8 m (approximately 15 steps) ahead of the point indicated by the A-GPS. This way, users know in advance the instruction, they have sufficient time to plan the upcoming action, and perform it safely with the assistance of the primary aids and the environmental cues. Figure 8 depicts this strategy.

#### 3.2.2. Participants

The following experiment complies with Universidad Panamericana policy on the ethics of research involving human subjects with disabilities.

Two blind male adults aged 34 and 38 participated in the experiment. The younger participant (subject A) had been blind since birth while the older (subject B) developed retinitis pigmentosa in his infancy. Subject B is considered legally blind due to the severe loss of vision. None of them reported any (known) disability in their sense of touch, feet, or cognition. Both reported more than 15 years of experience using the primary aids, particularly the white cane, therefore they were considered expert users.

Both subjects had already participated in a similar urban navigation experiment with a previous version of the AT device [46]. Nevertheless, they can be considered novel users: there is a three-year window between those experiments and the ones herein reported. As experimentally verified in [47], tactile feedback is retained in short-time memory. After just a month without any refresh or practice, people are uncapable of remembering tactile patterns.

#### 3.2.3. Procedure

Two urban environments in the downtown area of the city of Aguascalientes, Mexico were selected for the experiment. These settings are characterized for exhibiting an important affluence of people and vehicles.

Two paths comprising 280 and 600 m walking distances were fixed. Along these paths, acoustic traffic lights for enabling blind pedestrian street crossing were encountered together with static/dynamic obstacles caused by objects and other people.

A research assistant walked 5 to 10 m ahead of the subject for preventing and intervening in case of an unexpected risky situation.

Both environments were navigated by the subjects on the same day. Subject A performed the test one day early morning while subject B went through it three days later during the afternoon. The subjects never met.

#### 3.2.4. Method

Before the test, the two subjects completed experiment I at their own homes to get used to the tactile feedback and get familiar with the instructions provided by the user interface.

**Figure 9:**
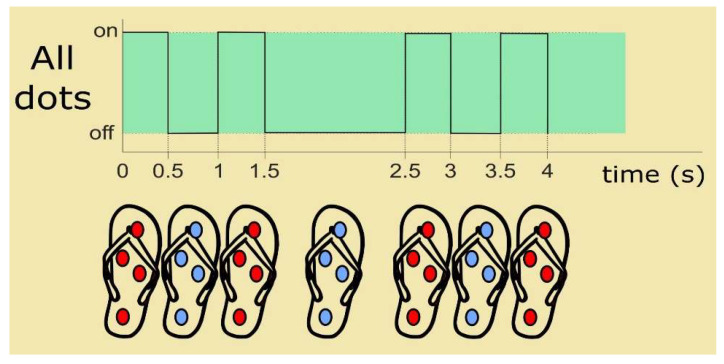
Vibrating pattern for stop.

A fifth instruction for indicating to stop (S) was added to the set of navigational instructions. It was encoded by setting the four dots simultaneously in two consecutive short sequences, a pause, and again two short sequences (like the typical SMS alert pattern in mobile phones (Figure 9). Satisfactory recognition rates were obtained for both subjects.

Subjects were then transferred by car to the chosen settings, which were completely unknown to them. The target destinations were set in the app by the research team. The smartphone was inserted into a rigid case which in turn was clipped to their belt. The smartphone was oriented towards the user’s front (see Figure 1) and was connected to the 4G network (Movistar, Telefónica México). Subjects were requested to remove both shoes and use the slippers. The right one contained the user interface. The electronic module was attached to the ankle accordingly.

Subjects were requested to walk in the direction indicated by the user interface. It was explained that the instructions are displayed in advance. They were asked to use the white cane and their mobility skills as they would do in a regular walk. They were aware that an assistant was nearby and that they could request any help if needed.

For monitoring purposes, the smartphone’s screen displaying the navigational app activity was mirrored to a tablet. The evolution of the subjects in the environments (i.e., their A-GPS coordinates and navigational times) were recorded.

#### 3.2.5. Results

**Figure 10:**
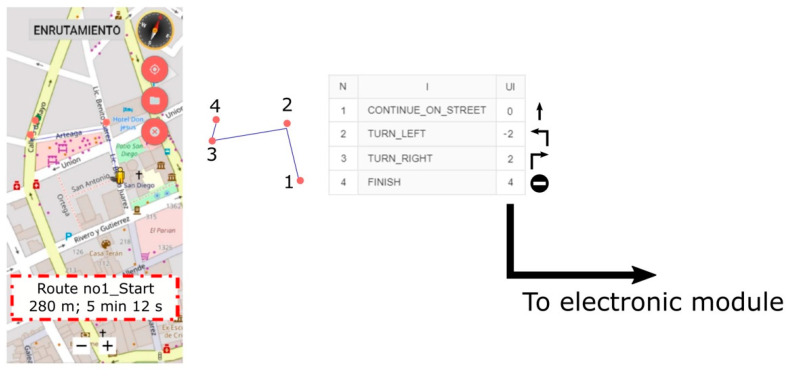
The first environment proposed to the subjects. Four nodes (orange circles) are needed to reach the destination. Table: the app assigns an instruction (column I) to each node (column N). The user interface number (column UI) associated to each instruction is sent to the electronic module when the user approaches the node. Instructions for “continue_on_street” and “finish” are F and S, respectively.

Figure 10 shows the first environment (E-1) proposed to the subjects. The task consisted of guiding the subjects along a 280 m path from the forecourt of a church to a well-known local store. Note that this path involves four nodes, i.e., the locations where the instructions are displayed.

The app builds a list assigning an instruction to each node. Each instruction has a user interface (UI) number that is transmitted to the electronic module when approaching the node (8 m before reaching it). The microcontroller interprets the UI number to display the corresponding tactile pattern.

**Figure 11:**
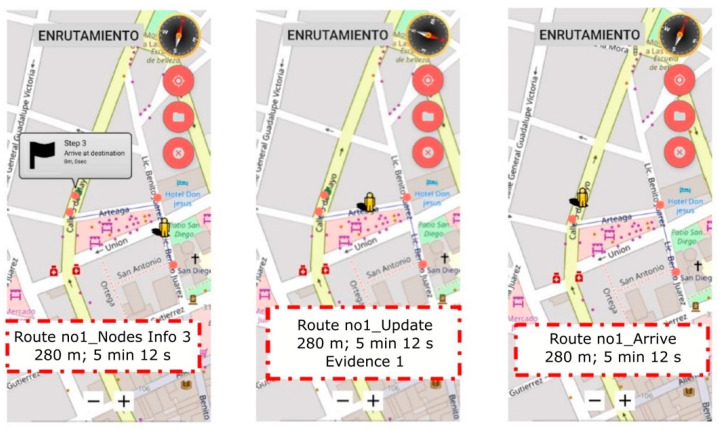
Subject A in E-1.

Figure 11 shows, for example, the progress of subject A in the environment. The task was successfully completed with no errors.

**Figure 12:**
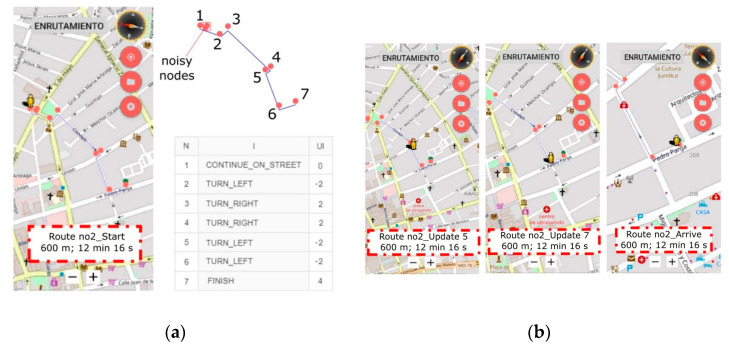
The second environment proposed to the subjects: (**a**) the path involved seven nodes and two noisy nodes that were neglected. (**b**) Some snapshots of subject B during the task.

Figure 12 shows the second environment (E-2) proposed to the subjects. The task consisted of guiding the subjects along a 600 m path from a random street point to a laundry establishment. Nine nodes were initially delivered by the GH API submodule (Figure 12a). However, the navigation software found that two of them were in a radius of less than 2 m and that neglecting them had no impact on the task. Therefore, these “noisy nodes” were eliminated from the final instruction list.

Figure 12b shows the progress of subject B in the environment. As in E-1, the task was successfully completed with no errors.

Table 3 summarizes the navigation times recorded from both subjects and the one estimated by the app for each environment. Note that the difference between subjects is negligible.

To determine the A-GPS accuracy, the subjects’ registered coordinates were compared to the coordinate datasets from OSM, which were assumed as the subjects’ actual positions. To align the two datasets, the Dynamic Time Wrapping (DTW) algorithm [48] was first applied. Then, the root middle square error (RMSE) was used to quantify the difference between both datasets.

**Figure 13:**
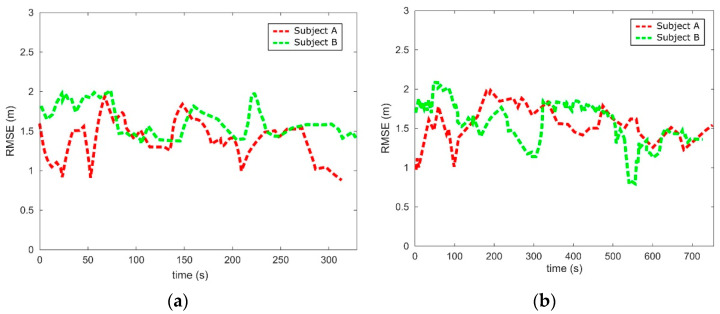
The A-GPS accuracy observed during the urban mobility task: (**a**) E-1 and (**b**) E-2.

The RMSEs observed during the navigation tasks are shown in Figure 13. Figure 13a shows the time-evolution of the RSME for E-1. An approximate 0.9 to 2 m A-GPS accuracy was observed during the task. Figure 13b shows the time-evolution of the RSME for E-2. A similar A-GPS accuracy range was obtained. Note that, despite having performed the same task with the same GPS receiver and 4G network, the time-evolution of the RMSEs is notably different between the subjects. This is due to the satellites position and atmospheric conditions that might have changed deriving from performing the task in different days.

**Figure 14:**
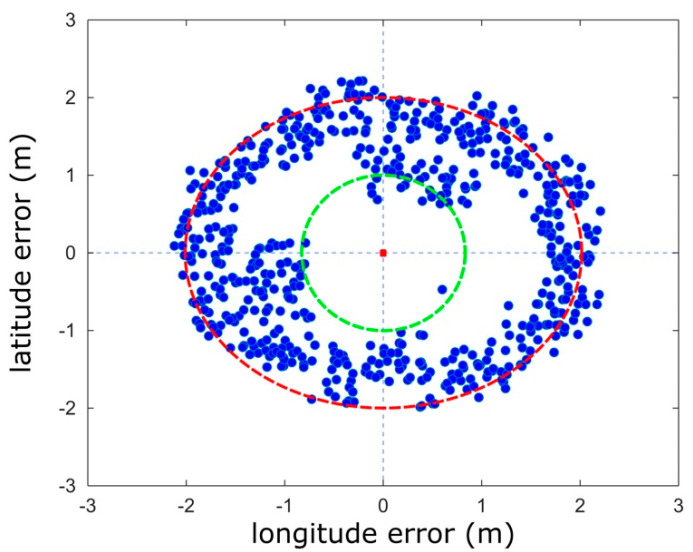
Scatter plot of the position error for the A-GPS coordinates.

Figure 14 shows the scatter plot of the position error for the ensemble of the A-GPS coordinates registered during the navigation tasks. The experimental data show that 95% of the A-GPS coordinates is in the accuracy range of 0.9 to 2 m.

During the task, the subjects encountered the typical urban obstacles such as street poles, trees, trash bins, cracked sidewalks, and other people. They easily surpassed them using the white cane. The subjects also had to wait for the acoustic green light to cross some streets.

We noticed that subjects stopped walking when the user interface displayed the tactile patterns. Overall, a natural fearless and doubtless walking was observed along the paths.

A post-experiment interview confirmed the intuitiveness of the information displayed and the low cognitive load demanded (instructions were displayed only when an action, i.e., a change of direction was required. Most of the journey the user interface did not display any information). Subjects stated that having the instructions displayed in advance is practical.

Some minor points for further improvement include trying to make the tactile patterns even shorter and to display the ‘forward’ instruction during long straight paths to avoid the sensation that the AT device is no longer working and to confirm the user that he/she is still walking in the correct direction.

The results obtained are undoubtedly encouraging. They confirm that A-GPS data is pertinent for the task and that the navigational software and the user interface are operational. Therefore, it is feasible to support the outdoor mobility of blind and visually impaired pedestrians with the proposed approach.

## 4. Conclusions

This paper has presented our progress and advancements in the design, implementation, and experimental evaluation of a novel Assistive Technology (AT) device devoted to support the urban mobility of blind and visually impaired pedestrians.

To offer a complete AT solution to the mobility challenges of visually disabled people, the proposal encompasses the primary aids for the obstacle detection process and addresses the orientation one with augmented GPS (A-GPS) data provided by a smartphone. User tracking and guidance in the environment is achieved by a dedicated Android-based app that locates the user on a map, computes the optimal route of travel, and generates the navigational instructions to reach a destination. Instructions are conveyed to the user via vibrotactile patterns that stimulate the foot sole, thus avoiding the traditional audio feedback that might compromise user attention in key moments during urban navigation.

Two experiments were conducted to evaluate the AT device. The first one verified the interface’s ability to transmit navigational instructions to the user and the user’s comprehension level to this feedback. The results showed very high recognition rates. The second experiment examined the AT device performance in real urban mobility with end users (blind pedestrians). The results showed that the AT device is capable of guiding users to a destination by providing the pertinent navigational instructions.

The proposed approach exhibits some interesting features: (1) wearability, (2) sound-free and hands-free operation, (3) inconspicuous and unnoticeable usage, (4) intuitive and fast to understand haptic feedback, (5) short learning and practice times to master its operation, (6) efficient and reliable performance, and (7) low-cost.

The AT device herein presented is in TRL (technology readiness level) 6 – technology demonstrated in relevant environment. Our current work focuses on the technology transfer process to a relevant stakeholder.

Future work will explore a tactile rendering strategy that prevents users from stopping their walk each time an instruction is displayed. It has also been envisaged to incorporate a remote monitoring functionality [49] into the navigation app that allows families and caregivers to locate the visually disabled pedestrian. This module will certainly increase the user’s safety.

## Figures and Tables

**Table 1:** AT for blindness and low vision: main research topics.

AT for blindness and low vision	Reading	Braille books	
		Audio books	
	Computer access	Speech synthesizers	
		Screen magnifiers	
		Braille terminals	
	Mobility	Obstacle detection	
		Orientation	Indoors
			Outdoors

**Table 2:** Navigational instruction recognition rates with the user interface and the proposed tactile rendering approach.

		Answered (%)			
		Forward	Backward	Left	Right
Presented	Forward	100	0	0	0
	Backward	0	100	0	0
	Left	0	0	95	5
	Right	0	7	0	93

**Table 3:** Experiment 2: Navigational times for the two environments.

	E-1 (280 m)	E-2 (600 m)
Subject A	5 min 12 s	12 min 35 s
Subject B	5 min 28 s	12 min 16 s
App	4 min	8 min
